# Health Tract, No. 175

**Published:** 1863-09

**Authors:** 


HEALTH TRACT, No. 175.

### SUMMER MORTALITY.

July and August are the most fatal months of the year in New-York, and other large cities. The deaths of August are nearly double those of November. This indicates that causes of disease are present in midsummer which are not found to the same extent in colder weather. Many attribute the increased mortality to unripe, imperfect, and decayed fruits; especially, as the deaths in charitable institutions, where fruits perfect or imperfect can not be indulged in, there are but half the number of deaths that occur in January and February; but in 1855, according to Commissioner Moreton's official report, one half of all who died were children under two years of age; such children are not those that use any kind of fruits much, hence the use of fruits has no appreciable influence on the greater mortality of summer. This, with the fact so reported, that there are more suicides in summer than in winter, the result of a diseased mind, induced by a diseased body, and that other fact, that the most incurable forms of consumption originate in summer, all combine to show that circumstances connected with warm weather are the direct causes of increased sickness and death in summer.

The most all-pervading cause of the increased sickness and death in cities in warm weather, is the breathing of an impure, a vitiated atmosphere. The most uncultivated know that there are "smells" connected with places in summer, which are not noticeable in winter. Many persons aim to have the rats about the house killed with poison, before the warm weather comes on, so as to avoid noisomeness about the premises. Hence, it must be set down as a practical fact, that warm weather generates odors which make the air impure; the breathing of which will always induce disease sooner or later, and more or less fatal, according to the degree of impurity and the duration of exposure to it. As double the number of persons die in the crowded parts of the city compared with less condensed districts; and as the poorer people are, the more crowded are their habitations, and poverty, and filth, and squalor, and uncleanness go together always and everywhere, it is proof positive that hot weather acting upon unclean habitations and surroundings, and thus vitiating the atmosphere, is the great overshadowing cause of the premature death and wasting sickness which pervades cities in summer-time. The practical inference is, that to prevent much of these calamities, all that is necessary is to secure a greater degree of cleanliness in person, in the houses, cellars, kitchens, back-yards, streets, and gutters.

Another cause of the greater mortality of summer is irregular, unseasonable, and over-hearty eating. If children especially are allowed to be nibbling at something all the time, the stomach is kept incessantly at work, until its strength is exhausted, as would be the case with any other muscle or set of muscles which were allowed no rest; when the stomach is thus weakened, or by taking more food into it than it can digest, or by eating heartily when very tired, the food sours; wind is formed, and the whole mass eaten is thrown up, or is passed out of the system, inducing diarrhea, cholera, or dysentery. While half of all who die in summer in the city are children under two years of age, over half of these children are under one year; and as the main food of such is mothers' or cows' milk, it is reasonable to infer, either that the children are fed too much on milk, or that it is not fresh and pure, is not perfect milk. The undoubted cause of the remarkable diminution of sickness and death among children in charitable institutions in New-York, can be from nothing else than the perfect cleanliness of these establishments, plainness of food, and regularity in eating and sleeping—a most suggestive statement to every parent.

